# Cephalo-medullary nailing versus dynamic hip screw with trochanteric stabilisation plate for the treatment of unstable per-trochanteric hip fractures: a meta-analysis

**DOI:** 10.1186/s13018-020-02193-5

**Published:** 2021-01-11

**Authors:** Amr Selim, Nikhil Ponugoti, Ali Zain Naqvi, Henry Magill

**Affiliations:** 1 Royal Cornwall Hospital, Truro, UK; 2 Cairo University, Cairo, Egypt; 3 Hillingdon Hospital, London, UK; 4 Chelsea and Westminster Hospital, London, UK

**Keywords:** Hip fractures, Unstable trochanteric fractures, Cephalo-medullary nail, Dynamic hip screw, Trochanteric stabilisation plate

## Abstract

**Background:**

The use of cephalo-medullary nails (CMN) is a widely accepted management option for the treatment of unstable per-trochanteric hip fractures. A growing body of literature has reported good functional and radiological outcomes in patients managed with a dynamic hip screw supplemented with a trochanteric stabilisation plate (DHS w/ TSP). However, a robust meta-analysis does not exist in the current literature comparing the two fixation methods.

**Purposes:**

Management of these kinds of injuries is very challenging in orthopaedic practice, yet no strong evidence is in place to delineate which implant gives the best results. This meta-analysis is the first to determine the efficacy of CMN versus DHS w/ TSP.

**Methods:**

An up-to-date literature search was performed using a predetermined search strategy and eligibility criteria. All suitable literature was appraised for methodological quality using the Cochrane’s collaboration tool. Hospital stay, operative time, intra-operative complication rate, mechanical failure rate, infection rates, revision rates and functional outcomes were all considered.

**Results:**

A total of five studies were included in the meta-analysis. The results of this analysis suggest that CMN is only associated with lower revision rates when compared to DHS w/ TSP; however, no significant difference was found in terms of hospital stay, operative time, blood transfusion, complications rate and functional outcome.

**Conclusion:**

Both CMN and DHS w/TSP proved to be reliable in the management of unstable per-trochanteric fractures; however, more extensive datasets are required to draw robust conclusions.

## Introduction

Hip fractures are one of the most common injuries affecting the elderly population and are associated with significant morbidity and mortality [1]. Fifty per cent of all hip fractures are per-trochanteric [2], of which up to 40% are considered unstable [3]. Per-trochanteric fractures occur in the area of the proximal femur between the intertrochanteric line and an imaginary horizontal line passing through the lower margin of the lesser trochanter. They are widely classified into stable and unstable fractures based on the fracture pattern [4].

The choice of management of unstable trochanteric fractures includes cephalo-medullary nails (CMN), dynamic hip screw alone or with the addition of a trochanteric stabilisation plate (DHS w/ TSP), proximal femoral locked plate (PFLP) and angular blade plates (ABP) [5]. Cephalo-medullary nails have been acknowledged and validated by many studies as reliable fixation choices for unstable trochanteric fractures [6–8]. Accordingly, the use of CMN as the primary management option for fixation of unstable per-trochanteric fractures is widely accepted [6, 9, 10]. Nonetheless, many studies have reported good functional and radiological outcomes in patients managed by DHS with the add-on TSP [5, 11, 12].

The biomechanical rationale of using the CMN in unstable trochanteric fractures is that the weight-bearing force acts through a shorter lever arm from the centre of hip rotation, thereby placing less stress on the implant [13]. Some authors report the intramedullary buttress effect as an additional factor in resisting fracture collapse with CMN [14]. On the contrary, many studies defend the use of DHS w/ TSP in these fractures because they provide a lateral wall buttress preventing excessive fracture varus collapse or medialisation [5, 15]. A further advantage of the TSP is the ability to apply this intraoperatively when DHS fixation was initially planned but subsequently deemed unsuitable as a sole fixation method.

Evans broadly classified per-trochanteric fractures into two types based on the fracture configuration [16]. In type I, the fracture line passes superiorly and laterally from the lesser trochanter, whilst type II includes those with fracture line passing inferiorly and laterally from the lesser trochanter. Furthermore, he subdivided type 1 fractures into 5 sub-types according to the fracture pattern. Unstable fracture patterns are described as those with loss of posteromedial support (type IC), lateral wall comminution (type ID), 4-part fractures (type IC) or with subtrochanteric extension (type 2). The Arbeitsgemeinschaft für Osteosynthesefragen/Orthopaedic Trauma Association (AO/OTA) has recently classified per-trochanteric fractures into three groups: 31A1 are simple per-trochanteric, 31A2 are multi-fragmentary per-trochanteric and 31A3 are subtrochanteric fractures [17]. AO/OTA 31A2 and 31A3 are considered unstable fractures.

Historically, PFLP and ABP were used as primary options in managing these fractures; however, their use diminished due to the high failure rate and poor functional outcome [5, 18, 19].

The literature suggests good outcomes for the management of unstable trochanteric fractures with both the CMN and DHS with the add-on TSP. However, no adequate or robust conclusions can be made from any of the individual studies available. We hereby conduct a meta-analysis to comprehensively analyse the outcomes of unstable trochanteric fractures managed by CMN versus DHS w/ TSP.

## Methods

### Literature search

The Preferred Reporting Items for Systematic reviews and Meta-analyses (PRISMA) [20] and the Cochrane Handbook for Systematic Review of Intervention [21] were implemented. We have searched both MEDLINE and Embase databases up to June 2020. The search was performed on the following 3 areas: “Dynamic Hip Screw” [Mesh] or “Trochanteric plate” [Mesh], and “Femoral nail”.

### Searching other resources

A further search was performed for any other previously published, planned and on-going trials by identifying references in ClinicalTrials.gov (http://clinicaltrials.gov/) and the World Health Organisation (WHO) International Clinical Trials Registry (http://apps.who.int/trialsearch/).

### Inclusion and exclusion criteria

The titles, abstracts and full text of articles that were deemed suitable for screening were reviewed by two of the study’s authors (AS and AZN). Any conflicts regarding the choice of included studies were determined by consensus amongst all study co-authors.

Inclusion criteria:

1. Level I, level II, level III (prospective and retrospective comparative studies) evidence;
2. Studies directly comparing CMN to DHS w/ TSP;
3. Subjects with per-trochanteric hip fractures only;
4. Subjects above 60 years of age; and
5. Human research.

Exclusion criteria:

1. Cadaveric or animal studies;
2. Articles primarily evaluating biomechanical properties of each fixation;
3. Articles primarily assessing radiological alignment of each fixation;
4. Abstracts, case reports, case series, letters and conference articles;
5. Subjects with open injuries; and
6. Studies with insufficient data.

### Outcome measures

The primary outcome measures of interest were as follows:

1. Hospital stay (days)
2. Operating time (minutes)
3. Blood transfusion required (units)
4. Complication rate
  a) Intraoperative complication rate
  b) Mechanical failure rate
  c) Infection rate
5. Revision rate
6. Function outcome
  a) A “good” functional outcome
  b) A “poor” functional outcome

### Data extraction

All primary outcome data was extracted by two independent authors, with no discrepancies present (AS and AZN). Study characteristics were extracted and recorded in Table 1. This includes first author, country, study design, level of evidence, number of patients (CMN; DHS w/ TSP), age of population (mean years), gender distribution (male to female; CMN; DHS w/ TSP), length of follow up (months), classification system and used functional score.

**Table 1:** Characteristics of included studies

Author	Country	Design	Level of evidence	No. of patients (CMN/DHS w/ TSP)	Age (mean)	Gender (M:F)	Follow-up	Classification system	Used functional score
Klinger 2005 [22]	Germany	Retrospective cohort	III	122/51	74	61/112	17	AO/OTA	The Merle d’Aubigné Hip Score
Madsen 1998 [11]	Norway	RCT	II	50/85	78.1/78.9	50/85	6	Evans and Zickels	Walking assessment (unaided, aided, unable)
Muller 2019 [23]	Germany	Retrospective cohort	III	200/100	82.6/83.6	200/100	24	AO/OTA	NR
Nuber 2003 [24]	Germany	Retrospective cohort	III	65/64	81/82	NR	6	AO/OTA	The Merle d’Aubigné Hip Score
Patil 2017 [25]	India	Prospective cohort	III	22/22	61.05	26/18	6	AO/OTA	Harris Hip Score

*CMN* cephalo-medullary nail, *DHS* dynamic hip screw, *TSP* trochanteric stabilisation plate, *M* male, *F* female, *No*. number, *RCT* randomised controlled trial, *NR* not recorded, *AO/OTA* Arbeitsgemeinschaft für Osteosynthesefragen/Orthopaedic Trauma Association

## Data synthesis and statistical analysis

Review Manager 5.4 was used for data synthesis and analysis. All continuous outcome data were evaluated using mean difference between the CMN and DHS w/ TSP groups. All discrete data were assessed by determining the risk ratio between the CMN and DHS w/ TSP groups. *P* values were calculated and documented for each outcome measure.

For each primary outcome measure, a random-effects statistical model was applied to data sets with high levels of heterogeneity between the studies. Heterogeneity was determined by *I*^2^; this indicates the percentage of variance attributable to the study heterogeneity. Zero to 25% indicates a low heterogeneity, 25–75% indicates moderate heterogeneity and > 75% suggests high levels of heterogeneity. The final analysis for each outcome measure is displayed in a forest plot with the accompanying confidence intervals (CI).

### Methodological quality assessment

Two authors (AS and AZN) independently assessed the quality and the accompanying risk of bias of all studies included in the meta-analysis. Randomised controlled trials (RCT) were evaluated via the Cochrane Handbook for Systematic reviews and intervention tool [21] dictated by the following parameters:

1. Randomisation
2. Concealment of allocation
3. Blinding of participants
4. Blinding of outcome assessment
5. Incomplete outcome data
6. Selective outcome reporting
7. Other bias

All non-randomised studies were assessed via the Newcastle-Ottawa Scale [26]. This tool implements a star rating system that grades the study from 0 to 9, where six or more is considered as a high-quality study. Disagreement regarding the grade was resolved by consensus amongst all co-authors.

## Results

### Literature search results

**Fig. 1:**
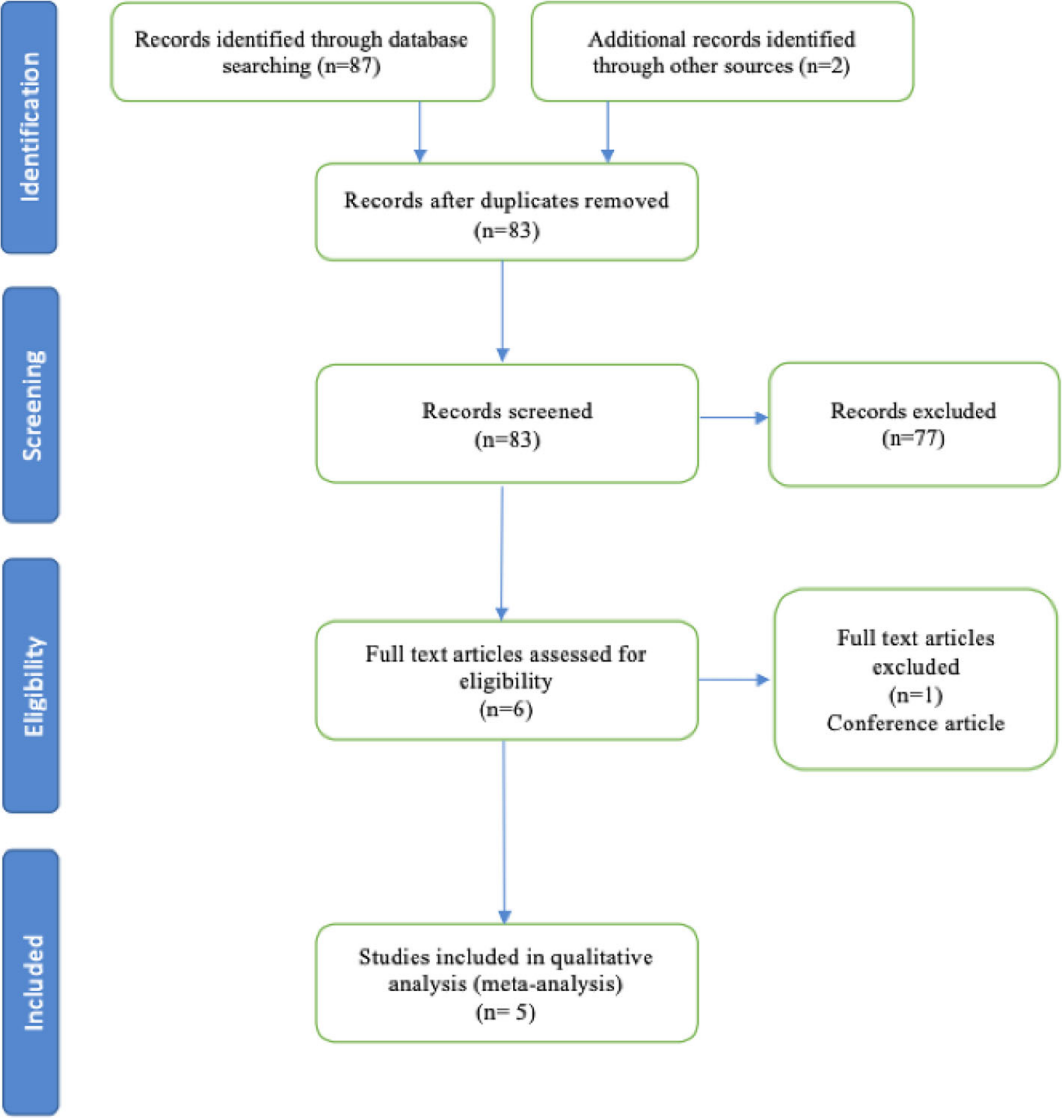
The Preferred Reporting Items for Systematic Reviews and Meta-Analysis

The initial search of the databases yielded 87 studies from MEDLINE and Embase; 2 additional suitable studies were added from other sources. Six duplicate studies were removed. Eighty-three remaining full-text articles were then screened, with 6 studies deemed suitable. One study did not meet the requirements of the inclusion criteria. Finally, 1 RCT and 4 cohort studies were deemed eligible for the meta-analysis. The PRISMA flow diagram and checklist for this search is shown in Fig. 1.

### Quality assessment

**Fig. 2:**
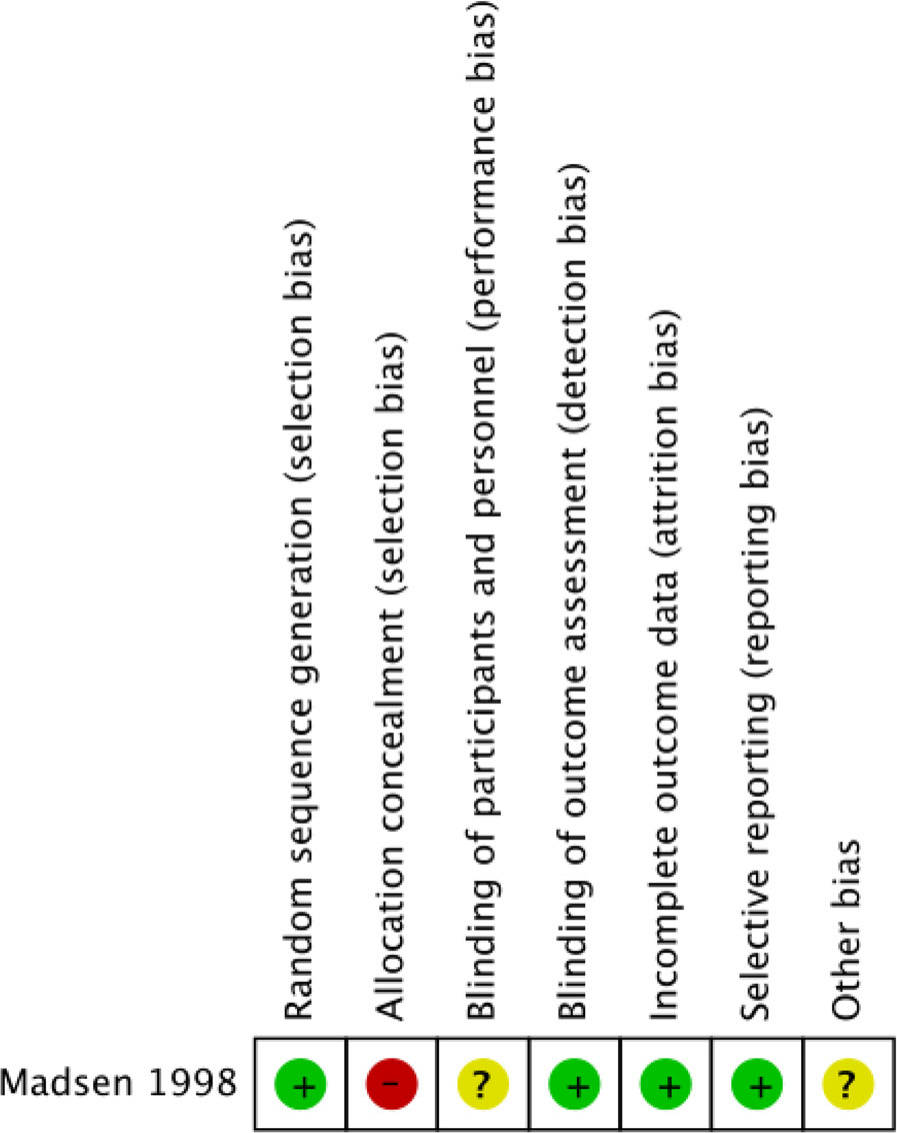
A figure displaying the risk of bias for the RCT included in the meta-analysis. Each colour represents the risk of bias in each of the domains (red = high risk, yellow = unclear and green = low risk)

The RCT included in the meta-analysis was assessed for bias according to Cochrane Handbook for Systematic reviews and interventions [11]. This paper was deemed to have low bias in terms of randomisation, blinding out outcome assessment, outcome data recorded and selective reporting. However, it was noted to have high levels of bias with regards to allocation concealment (Fig. 2). The remaining non-randomised studies were assessed for quality using the Newcastle-Ottawa Score with a subjective score out of 9. A good quality study has 3 or 4 stars in the selection domain and 1 or 2 in the comparability domain and 2 or 3 in the outcome/exposure domain. A table displaying the scores is shown in Table 2.

**Table 2:** A table displaying the methological index for non-randomised studies (Newcastle-Ottawa Scale). Selection (maximum 4), comparability (maximum 2) and outcome (maximum 3)

Study	Selection	Comparability	Outcome	Total score
Klinger 2005 [22]	3	2	3	8
Muller 2019 [23]	3	2	3	8
Nuber 2003 [24]	3	2	2	7
Patil 2017 [25]	3	2	2	7

### Characteristics of studies included

All included studies were published between 1998 and 2019. In total, the studies included 781 patients. A total of 459 subjects underwent fixation with CMN and 322 with DHS w/ TSP. The follow-up time for the involved studies ranged from 9 months to 50.4 months (mean 15.4). The details of the RCT and 4 cohort studies included in our analysis are reviewed in Table 1. Patterns of fractures in each study according to AO/OTA classification are included in Table 3.

**Table 3:** A table showing fracture types in each study according to AO/OTA classification system

Study		A2.2	A2.3	A3
Klinger	CMN	105		68
	DHS w/ TSP			
Muller	CMN	200		0
	DHS w/ TSP	100		0
Nuber	CMN	18	20	27
	DHS w/ TSP	24	32	8
Patil	CMN	5	12	5
	DHS w/ TSP	8	13	1

*CMN* cephalo-medullary nail, *DHS* dynamic hip screw, *TSP* trochanteric stabilisation plate, *AO/OTA* Arbeitsgemeinschaft für Osteosynthesefragen/Orthopaedic Trauma Association

## Outcomes

### Outcome 1: hospital stay (days)

**Fig. 3:**
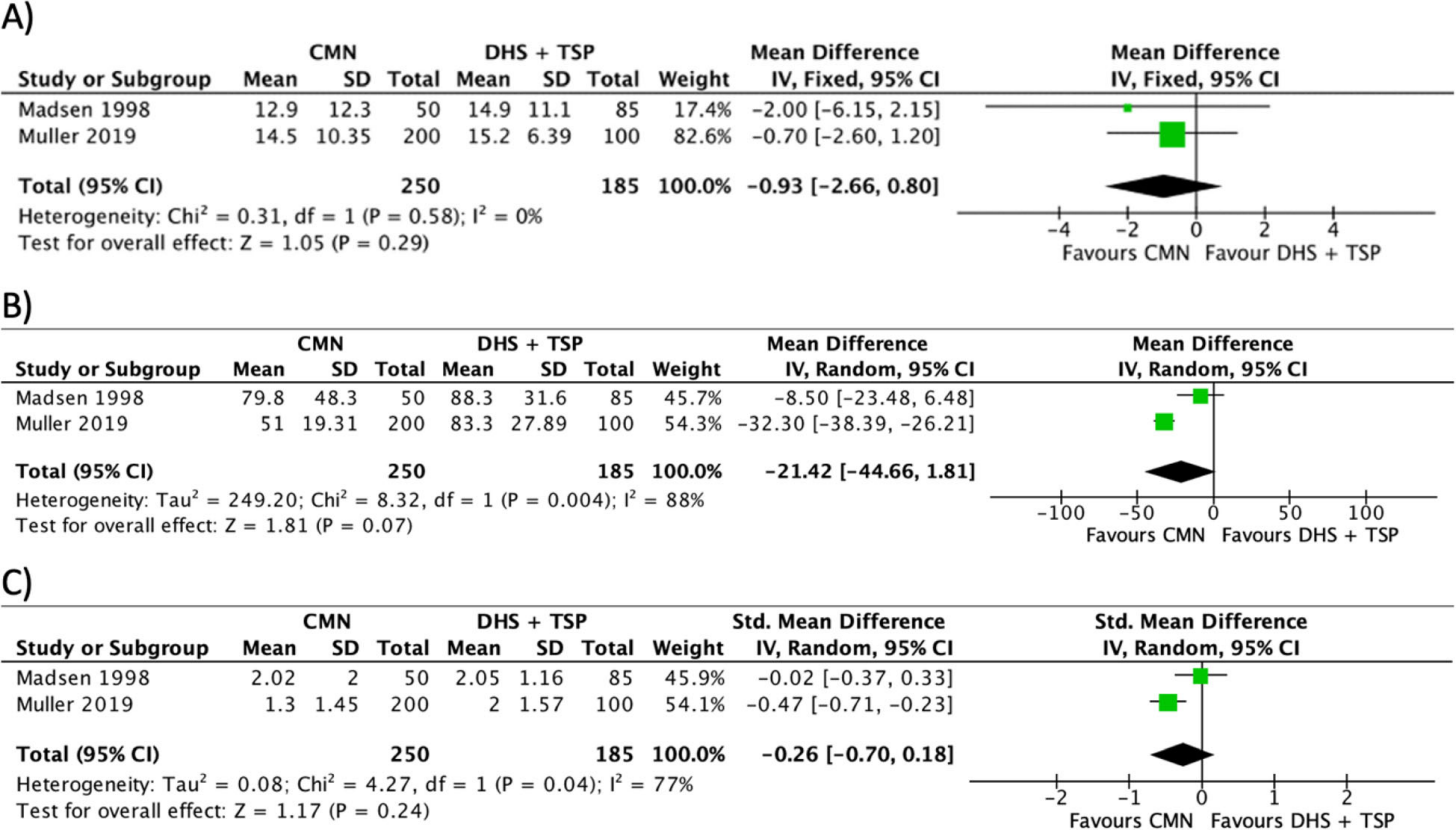
A forest plot showing the comparison of **a** hospital stay (days), **b** operative time (minutes) and **c** blood transfusion (units) between the two fixation methods. CI, confidence interval; IV, independent variable; M-H Mantel-Haenszel; CMN, cephalo-medullary nail; DHS, dynamic hip screw; TSP, trochanteric stabilisation plate

The hospital stay was reported in 2 studies (*n* = 435) with a low level of heterogeneity (*I*^2^ = 0%) [11, 23]. The difference between the CMN and DHS w/ TSP groups in terms of hospital stay is not statistically significant (Fig. 3a).

### Outcome 2: operation time (minutes)

The operation time was reported in 2 studies (*n* = 435) with a high level of heterogeneity (*I*^2^ = 88%) [11, 23]. When a fixed model is applied, the result favours CMN where operative time is significantly shorter. However, we applied a random effects model due to the high level of heterogenicity, and the difference becomes no longer statistically significant (Fig. 3b).

### Outcome 3: blood transfusion (units)

The number of transfused blood units was reported in 2 studies (*n* = 435) with a high level of heterogeneity (*I*^2^ = 77%) [11, 23]. When a fixed model is applied, the result favours CMN where the number of transfused blood units is significantly lesser. A random effects model is applied instead due to the high level of heterogenicity; accordingly, the difference is no longer statistically significant (Fig. 3c).

### Outcome 4: complication rate

**Fig. 4:**
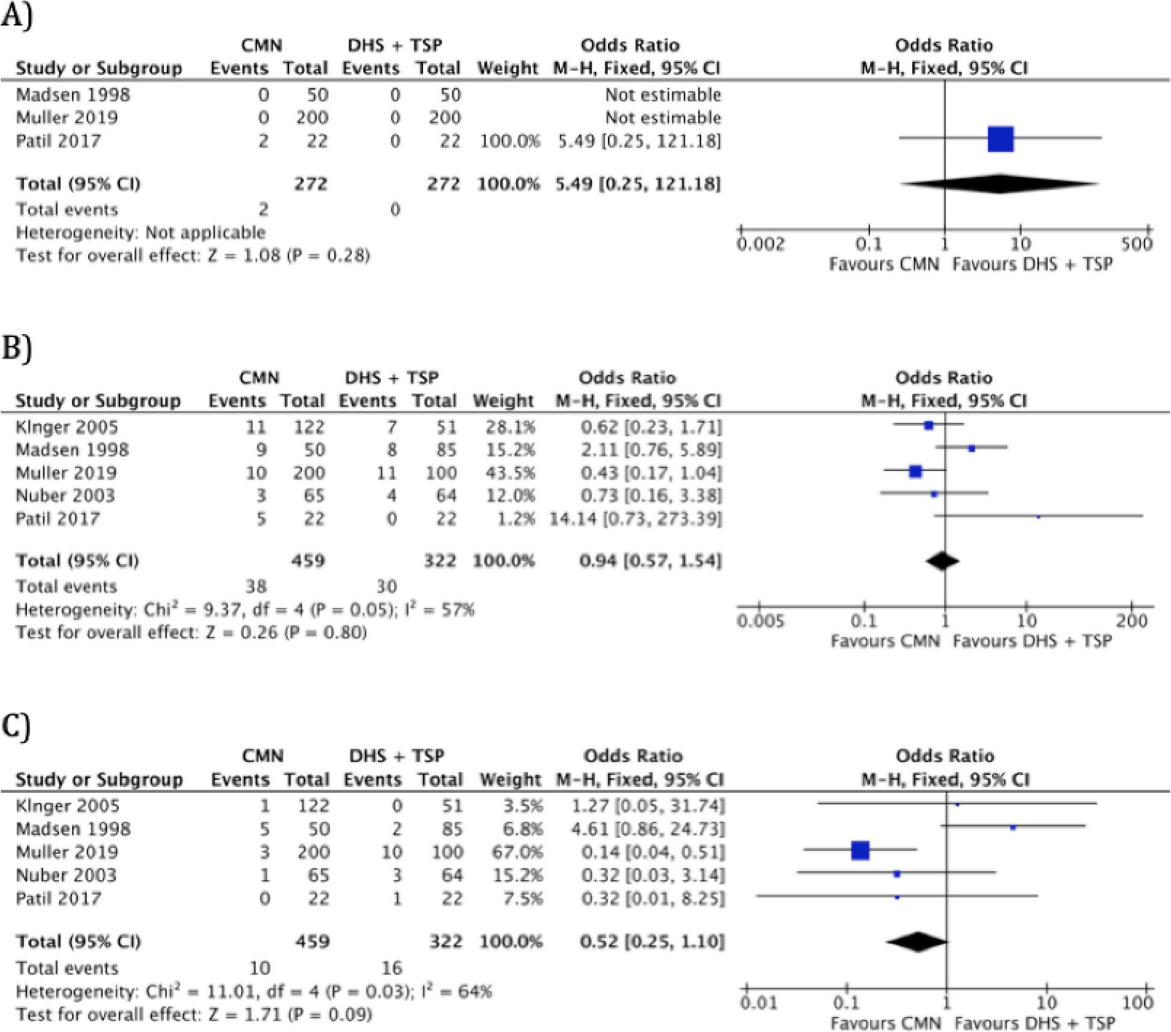
A forest plot showing the comparison of **a** hospital stay (days), **b** mechanical failure rates and **c** infection rates between the two fixation methods. CI, confidence interval; IV, independent variable; M-H, Mantel-Haenszel; CMN, cephalo-medullary nail; DHS, dynamic hip screw; TSP, trochanteric stabilisation plate

Intra-operative complication rates were reported in 3 studies (*n* = 544) [11, 23, 25]. Only 2 positive events were recorded across the data set [25]. The available data suggests no significance between the operative groups (Fig. 4a). Mechanical failure rates were reported in all 5 studies (*n* = 781) with a moderate level of heterogeneity (*I*^2^ = 57%) [11, 22–25]. The comparative analysis suggests no significance between the operative groups (Fig. 4b). Infection rates were reported in 5 studies (*n* = 781) with a moderate level of heterogeneity (*I*^2^ = 64%) [11, 22–25]. The comparative analysis suggests no significance between the operative groups (Fig. 4c).

### Outcome 5: revision rate

**Fig. 5:**
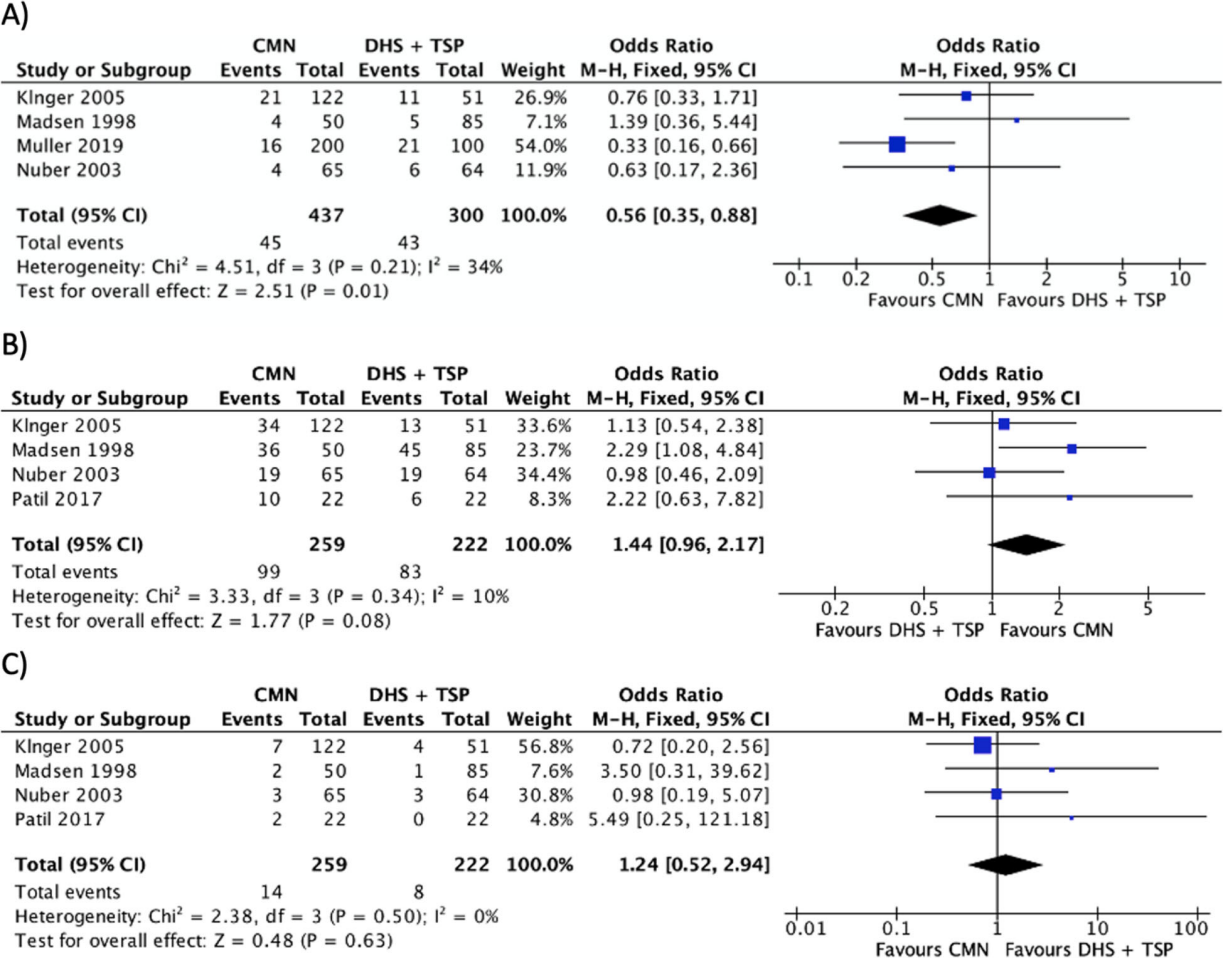
A forest plot showing the comparison of **a** revision rates, **b** “good” functional outcomes and **c** “poor” functional outcomes between the two fixation methods. CI, confidence interval; IV, independent variable; M-H, Mantel-Haenszel; CMN, cephalo-medullary nail; DHS, dynamic hip screw; TSP, trochanteric stabilisation plate

Revision rates were reported in 4 studies (*n* = 737) with a moderate level of heterogeneity (*I*^2^ = 34%) [11, 22–24]. The comparative analysis suggests that the CMN group was associated with significantly lower revision rate when compared to DHS w/ TSP (Fig. 5a).

### Outcome 6: functional outcome

Functional outcomes were reported in 4 studies (*n* = 481) with low to moderate level of heterogeneity (*I*^2^ = 0–29%) [11, 22, 24, 25]. Post-operative function was assessed using the The Merle d’Aubigné Hip Score in 2 studies [22, 24]. Patil et al. assessed subjects with the Harris Hip Score [25]. Madsen at al. assessed function with a walking assessment (Unaided, Aided, Unable) [11]. All scores were broadly categorised as “poor”, or “good” to achieve consistency in the forest plot analysis. A good functional score corresponded to the “excellent” category in Merle d’Aubigné and Harris Hip Scores, and “unaided” category in walking assessment. On the other hand, poor functional score involved “poor” category in Merle d’Aubigné and Harris Hip Scores, in addition to “unable” category in walking assessment.

The comparative analysis suggests no significance between the operative groups in terms of good functional outcome (Fig. 5b) or bad functional outcome (Fig. 5c).

### Sensitivity analysis

A sensitivity analysis was performed on all statistically significant results. If a random effects model was applied for the comparison of operative times, transfusion units required and revision rates, the result was no longer statistically significant. All other statistical conclusions remain unchanged after sensitivity analysis.

## Discussion

To the best of our knowledge, this is the first meta-analysis to compare the cephalo-medullary nail and dynamic hip screw with trochanteric stabilisation plate for the treatment of unstable per-trochanteric hip fractures.

Few meta-analyses exist in literature comparing DHS alone to CMN for the management of these kinds of fractures [6, 27, 28]. However, most of the recent research demonstrated superior results of CMN to DHS alone when it comes to unstable trochanteric hip fractures management [6, 8, 27–29].

Failure in cases managed by DHS alone is believed to be due to excessive lag screw sliding with collapse or medialisation of the distal fracture fragment even if the lag screw is optimally positioned in the femoral head [6, 27, 28]. The theoretical reasons behind that are thought to be either loss of the calcar support or lateral femoral wall insufficiency resulting in fracture collapse with loading [30].

The most significant finding in our study indicates that the CMN is only associated with lower revision rates when compared to DHS w/TSP.

Revision surgery was required in the CMN group for a variety of reasons: lag screw cut-out, deep infection, periprosthetic failure and non-union. Failures that justified revision in the DHS w/ TSP group were lag screw cut-out, deep infection, fracture displacement and non-union. These individual parameters were not consistently recorded in any of the included studies. We believe outcome measures such as union time, avascular necrosis and fracture alignment would provide useful comparisons and may correlate with long-term function or justify the difference in revision rates.

Despite this positive finding, the data in this meta-analysis demonstrate no significant difference in relation to length of hospital stay, operative time, blood transfusion, complications rate (intra-operative complication, mechanical failure and infection rate) and post-operative functional outcome. In the dataset, only two cases of intra-operative complications were that of “iatrogenic fracture while distal locking” [25]. The authors mentioned that cerclage wiring was used due to inaccessibility to long nails on that occasions and both patients had their fractures united uneventfully. “Mechanical failure” was a broad category that included lag screw cut-out, Z-effect, secondary displacement (with excessive medialisation or varus collapse) or peri-prosthetic fracture [11, 22–25].

Matre et al. conducted an important RCT which included 684 patients with trochanteric or sub-trochanteric hip fractures treated by TRIGEN INTERTAN CMN versus DHS. Seventy per cent of patients managed by DHS had also TSP applied. INTERTAN nails and DHS had similar outcomes in terms of pain, function and reoperation rates [31]. We did not include this study in our analysis because a different design of the standard IMN was used, in addition to inability to exclude patients managed by DHS alone from the outcome data. Moreover, it was not directly comparing between IMN and DHS w/ TSP which is one of our inclusion criteria.

A number of limitations in the present study should be highlighted. Only five studies exist in the literature that include a total of 781 subjects. The mean follow-up time is 15.4 months; this was as low as 6 months in three studies [11, 24, 25]. This may be inadequate in order to capture all cases that experience long-term failure or require revision surgery. Outcomes such as the intra-operative complications rate are serious, yet uncommon, and therefore larger subject numbers are required for an appropriately powered analysis.

One of the noted limiting factors is the difference in the implant design used across the studies. Four studies used the Proximal Femoral Nail Anti-Rotation (PFNA) design with 2 proximal screws [22–25] whilst in one study, the Gamma nail design with one proximal lag screw was utilised [11]. Additionally, it was not clearly mentioned whether they used the long or short nail designs, and the titanium or stainless-steel nail types. In regard to DHS w/ TSP, one study used a different TSP design [25], where the TSP was smaller and fitted from inside the DHS plate through the barrel, than the standard TSP used in the other four studies [11, 22–24].

We also appreciate that the majority of papers included are cohort studies and therefore may lower the quality of the data included. In addition, all cohort studies were inadequately randomised whereby “surgeon’s preference” was always stated as the reason for a choice of fixation method [22–25]. The blinding of both participants and surgeons remains an inherent impracticality of all studies. Despite this, all included studies were categorised as high quality across the vast majority of assessed bias domains.

## Conclusion

The results of this analysis suggest that CMN is only associated with lower revision rates when compared to DHS w/ TSP. The study demonstrates no significant difference in terms of hospital stay, operative time, blood transfusion, complications rate and functional outcome. Further literature, especially well-conducted RCTs, is required to draw robust conclusions and confirm long-term results.

## Data Availability

Data can be made available upon request to the corresponding author.
